# Study of Electrochemical Transformation of Anacardic Acid from Cashew (*Anacardium occidentale*) Nut Shell Liquid

**DOI:** 10.3390/molecules30061330

**Published:** 2025-03-16

**Authors:** Alvaro A. Arrieta, Jorge A. Ducuara, Yamid Nuñez de la Rosa

**Affiliations:** 1 Department of Biology and Chemistry, University of Sucre (Universidad de Sucre), Sincelejo CP 700001, Colombia; 2 Faculty of Engineering and Basic Sciences, Fundación Universitaria los Libertadores, Bogotá D.C. CP 111221, Colombia; jaducuarah@gmail.com (J.A.D.); yenunezd@libertadores.edu.co (Y.N.d.l.R.)

**Keywords:** electrochemical oxidation, anacardic acid, organic acids, cashew nut shell liquid, cyclic voltammetry, biomass valorization

## Abstract

The valorization of agricultural waste has gained attention due to the need for sustainable technologies addressing environmental and economic challenges. The aim of this work is to investigate the electrochemical transformation of anacardic acid, a major component of cashew nut shell liquid (CNSL), into organic acids and polymeric materials. Cyclic voltammetry (CV) was carried out on ethanolic anacardic acid solutions with NaOH as the supporting electrolyte to induce oxidation reactions. CV, FTIR spectroscopy, and electrochemical impedance spectroscopy (EIS) were used to analyze the transformation processes. The results revealed oxidation sequences involving phenoxyl radicals, hydroquinones, and benzoquinones, leading to ring-opening reactions and the production of low-molecular-weight organic acids, including propionic, formic, oxalic, lactic, and acetic acids, as confirmed by HPLC. Polymerization processes were also observed, leading to the attainment of polymeric materials. FTIR spectra showed changes in phenol and carboxylic acid bands, confirming chemical transformations. CV and EIS indicated irreversible oxidation processes with charge transfer coefficients between 0.397 and 0.414 controlled by diffusion–adsorption. This work demonstrates the feasibility of electrochemical oxidation as a green method for producing organic acids from anacardic acid, aligning with circular economy principles and reducing reliance on petrochemical sources. It highlights the potential of electrochemical approaches for sustainable biomass utilization and fine chemical synthesis.

## 1. Introduction

The utilization of agricultural waste has received increasing attention recently, driven by the need to develop sustainable technologies that help address ecological and financial challenges. For example, lignocellulose has been used to produce bioethanol, biodiesel by biochemical and thermochemical methods, as well as in the production of products such as xylose and biopolymers [1,2]. Residues from the coconut, rice and cashew agroindustry have been used to generate graphene materials and valorize the byproducts of these agroindustry [3,4]. A byproduct that has attracted attention in recent years is cashew nut shell liquid (CNSL), a derivative of cashew nut (*Anacardium occidentale*) processing. CNSL is an abundant and renewable source of phenolic compounds, among which anacardic acid is a prominent component.

**Figure 1:**
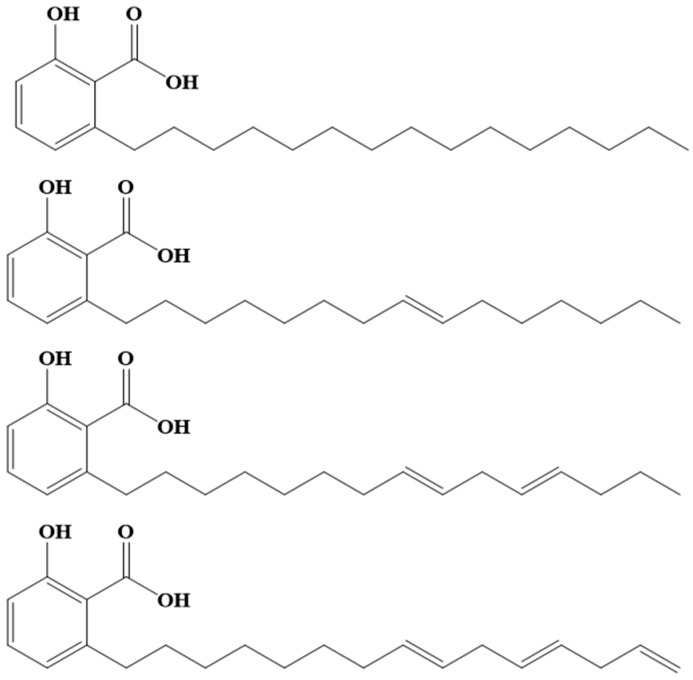
Molecular structure and composition of anacardic acid.

Anacardic acid is a mixture of salicylic acid derivatives with 15-carbon alkyl chains that can present various levels of unsaturation and constitutes approximately 70% of CNSL. A schematic of the chemical structure of anacardic acid is presented in Figure 1. This compound is recognized for its structural versatility and inherent reactivity, which makes it an ideal candidate for chemical modifications. The carboxylic acid, phenolic and unsaturation functional groups present in anacardic acid can undergo various reactions, allowing for its modification and chemical transformation. Anacardic acid has been used as an additive to a mixture of anacardic acid and chitosan to prepare polymeric biofilms for smart packaging with antimicrobial activity [5]. Anacardic acid has been used to obtain a biobased 4′,5′-dinitro anacardic acid copper (II) complex (2:1 form) and its application as a homogeneous catalyst for room-temperature synthesis of 1,4-napthoquinone [6]. An electroactive biopolymer of anacardic acid/cassava starch has also been synthesized, which showed oxidation/reduction processes [7]. Reviews on modifications and applications of anacardic acid can also be found [8,9].

On the other hand, organic acids, such as succinic acid, fumaric acid, and malic acid, among others, are substances of interest for the production of bioplastics, food additives, pharmaceutical products and biodegradable materials [10]. The traditional synthesis processes of some of the low-molecular-weight organic acids are commonly linked to raw materials of petrochemical origin, which generate a significant environmental footprint and a negative impact on ecosystems. The synthesis of these compounds by electrochemical methods offers an alternative route that is not only more sustainable, but also highly adjustable, allowing for precise control over the reaction conditions and the selectivity of the product. In this sense, the transformation of anacardic acid into organic acids is aligned with global efforts in environmental care and sustainable chemistry to move towards renewable resources and circular economies.

Electrochemical synthesis allows for redox (oxidation and reduction) reactions to be performed under mild conditions, with minimal use of hazardous reagents. These techniques use electric current to drive reactions, offering advantages such as sustainability by eliminating toxic chemical oxidants and reducers, scalability at both the laboratory and industrial levels, and selectivity by fine-tuning reaction parameters to favor desired products. Electrochemistry has been used to transform biomasses; for example, lignin has been degraded through the selective breaking of β-O-4 bonds by electrochemical oxidation, and adhesive precursors have also been produced by electrochemical oxidation of this biocomposite [11,12,13].

Electrochemical oxidation, in particular, has been explored to transform phenolic compounds into some chemical derivatives such as quinones and other derivatives [14,15]. Anacardic acid, with its phenolic and carboxylic functionalities, is particularly suitable to be transformed by electrochemical processes, presenting opportunities to generate high-value organic acids through a sustainable approach [14]. Conventional methods for transforming anacardic acid and other phenolic compounds typically require harsh chemical oxidants, high temperatures, and metal catalysts, often generating hazardous by-products and increasing environmental impact. In contrast, electrochemical oxidation offers a sustainable alternative, operating under mild conditions without external chemical oxidants, reducing waste generation, and aligning with green chemistry [5,6,8,9].

Studies have highlighted the role of CNSL derivatives in antimicrobial and anticorrosive applications, the catalytic conversion of CNSL into biofuels and industrial chemicals [8,16,17,18,19], as well as preliminary insights into the electrochemical transformation of CNSL [14,20,21]. However, an exploration of its electrochemical transformation into organic acids remains an understudied area. Addressing this issue is crucial to potentialize the use of CNSL as a sustainable feedstock.

This work aims to investigate the transformation by electrochemical oxidation of anacardic acid into organic acids and polymeric material using electrochemical techniques of cyclic voltammetry and electrochemical impedance spectroscopy, and FTIR spectroscopy to elucidate the possible reaction mechanisms and some electrochemical parameters involved in the oxidation processes. The findings of this work may contribute to the advancement of the field of electrochemical valorization of biomass-derived compounds. By focusing on a sustainable route for the synthesis of organic acids and a polymeric material that can be modified or enhanced for technological applications, this study aligns with the broader goals of reducing dependence on fossil fuels, minimizing waste, and promoting innovations in sustainable chemistry. Furthermore, it provides a framework for future studies on the electrochemical processing of complex biomass-derived compounds, paving the way for their integration into industrial applications.

## 2. Results and Discussion

**Figure 2:**
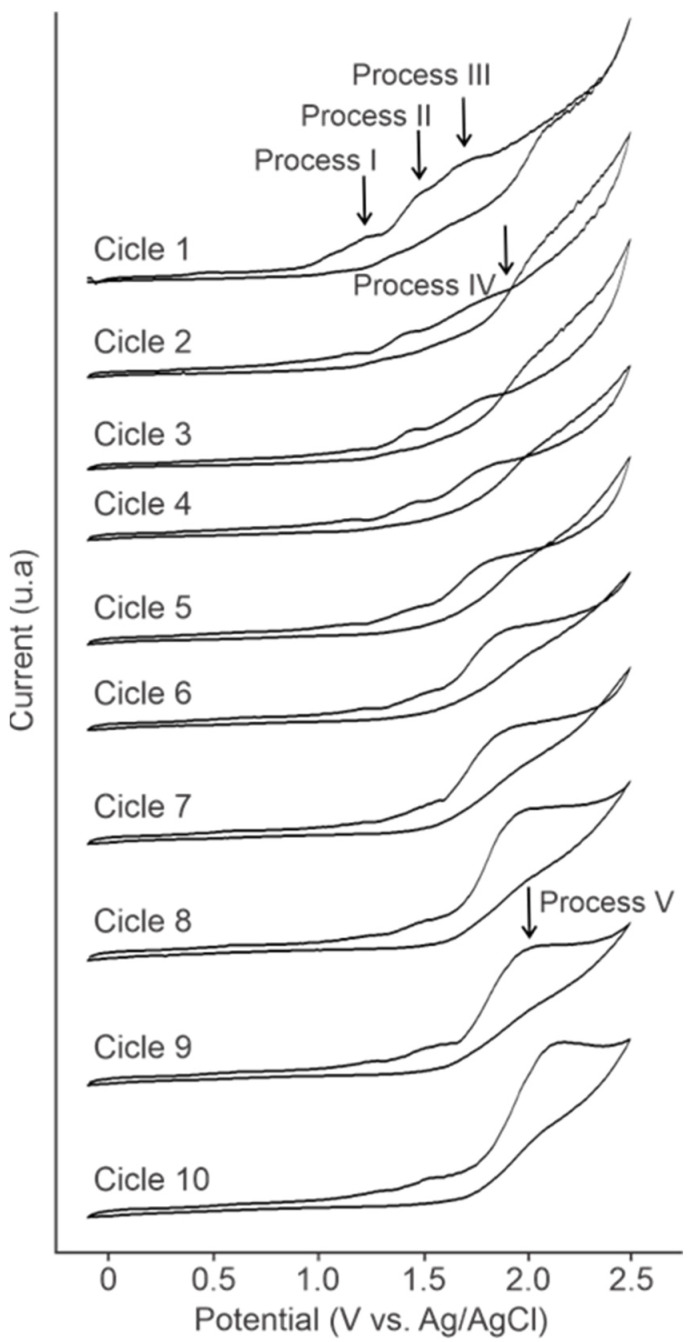
Succession of 10 voltametric cycles at 10 mV/s of anacardic acid in methanol and NaOH.

The initial experiments consisted in identifying the electrochemical processes of anacardic acid. Figure 2 shows 10 successive cycles of anacardic acid recorded at 10 mV/s in methanol solution with NaOH as supporting electrolyte.

In the first cycle, three irreversible oxidation processes of anacardic acid can be observed in the anodic wave. The first oxidation process takes place at 1.24 V and occurs in the ring of the phenolic group of the anacardic acid molecule, generating its conversion into phenoxy radicals, which undergo a second oxidative transformation at a potential of 1.49 V that generates the production of hydroxyquinones and benzoquinones. The formation of these intermediates from phenolic compounds and their electrochemical oxidation processes have been previously reported [22]. These compounds are transformed into low-molecular-weight organic acids due to the oxidation that occurs at 1.73 V that generates carboxyl groups. It has been reported in the literature that organic acids can be generated by ring and alkyl chain cleavage [22,23,24].

After the second cycle, a loop at 1.91 V is clearly observed due to the nucleation process of phenoxyl radicals, which react in an oligomerization or polymerization process. This nucleation loop moves to more positive potentials in the successive cycles until it disappears in the eighth cycle at 1.93 V, 2.01 V, 2.08 V, 2.35 V, and 2.36 V in cycles 3, 4, 5, 6, and 7, respectively. Although the potential change between cycles 2 and 3 is minimal (from 1.91 V to 1.93 V), this change becomes more evident in subsequent cycles, possibly due to progressive polymerization and changes in the electrode surface properties, which affect the nucleation overpotential. Furthermore, a reduction in the potential change is also observed in cycles 5 and 6 (2.35 V to 2.36 V), which may be related to a reduction in or depletion of the nucleation sites because the amount of polymer formed in these cycles is already appreciable.

From the eighth cycle onwards, a broad oxidation peak at 2.03 V is observed (process V) which may be due to oxidation processes of the fragments, generation of organic acids, and the growth or increase in the amount of polymer generated. The oxidation processes of phenol, the formation of intermediates and the polymerization process were reported in previous studies [24,25,26].

**Figure 3:**
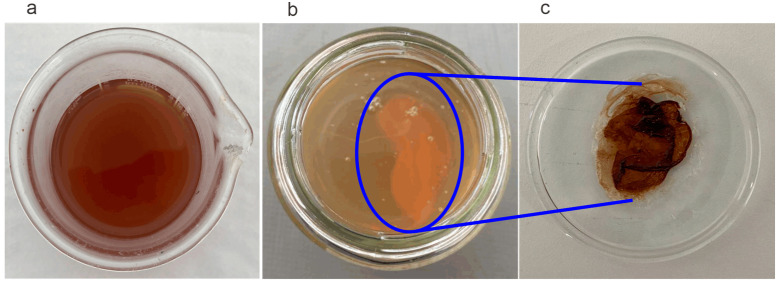
Image of anacardic acid before (**a**) and after (**b**) applying the electrochemical treatment and polymeric material (**c**).

Figure 3 shows the images of anacardic acid without electrochemical treatment (Figure 3a), the organic acid mixture solution resulting after electrochemical treatment (Figure 3b), and the polymeric material generated (Figure 3c). The initial anacardic acid solution is a dark-brown solution that, after electrochemical treatment, becomes a clear amber solution with a precipitate at the bottom that corresponds to the polymeric fraction formed.

**Figure 4:**
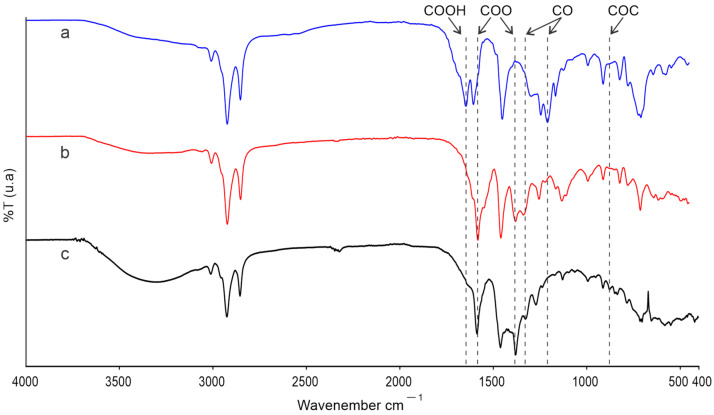
Infrared spectroscopy spectra of anacardic acid before (**a**) and after (**b**) electrochemical treatment, and (**c**) polymeric material.

The FTIR spectra of the anacardic acid sample without electrochemical treatment, after electrochemical treatment, and the polymeric material formed by the electro-oxidation processes are presented in Figure 4.

It can be observed that the spectrum of anacardic acid (Figure 4a) exhibits a broad band between 3675 and 3105 cm^−1^, associated with the vibrations of the OH groups, between 3031 and 2810 cm^−1^ and 1452 cm^−1^, the bands assigned to ArH (aromatic hydrogens) and the C-H of the aliphatic chains. In the region of 1648 cm^−1^ and 1605 cm^−1^, vibration of protonated carboxylic groups -COOH is observed. The bands at 1244 cm^−1^, 1208 cm^−1^, and 1165 cm^−1^ correspond to the vibrations of C-O. These results are consistent with those reported in the literature [27].

The spectrum of the solution resulting from the application of the electrochemical treatment (Figure 4b) exhibits a notable contrast with respect to the starting anacardic acid solution. However, it presents regions with similar bands due to the structural similarities of its components. The main differences are observed in the regions around 1645 cm^−1^, 1605 cm^−1^ and 1451 cm^−1^ because the band corresponding to the vibrations of the -COOH protonated (1645 cm^−1^) disappears; so, only two bands are observed, which shift to 1583 cm^−1^, which could be assigned to -COO- carboxylate groups [28], and 1458 cm^−1^ (related to the C-H of the aliphatic chains). A band appears in the region around 1338 cm^−1^ associated with the aliphatic C-H groups: 1379 cm^−1^ assigned to the -COO-, and 1131 cm^−1^ due to the C-O vibrations. The infrared spectrum of the polymer (Figure 4c) shows differences in some bands in relation to the spectra of anacardic acid before and after treatment, as well as differences in their relative intensities. This is due to the structural transformation. However, bands similar to those observed in the spectra of anacardic acid before and after treatment are also observed due to their structural similarities. The most marked differences are the increase in the OH vibration region, in the region of 3675–3105 cm^−1^, possibly due to the generation of hydroxyl groups in the ortho, meta and para positions in phenol, and 1584 cm^−1^ and 1374 cm^−1^, assigned to the -COO-. The appearance of a band at 1061 cm^−1^ and 878 cm^−1^ due to C-O-C vibrations may be due to the formation of a new skeletal structure, possibly related to C-O-C bonds in polymerization.

To confirm the presence of organic acids generated by electro-oxidation, the resulting solution after applying the electrochemical treatment (Figure 3b) was analyzed by HPLC chromatography. The results confirmed the presence of propionic acid (13.50 mg L^−1^), formic acid (34.64 mg L^−1^), oxalic acid (45.58 mg L^−1^), lactic acid (41.44 mg L^−1^), and acetic acid (65.99 mg L^−1^). The production of organic acids can vary not only in the quantities produced but also in their type depending on the experimental conditions, such as electrode type, electrode area, acid and electrolyte concentrations used, electrolyte type, electrochemical technique, and parameters employed.

**Figure 5:**
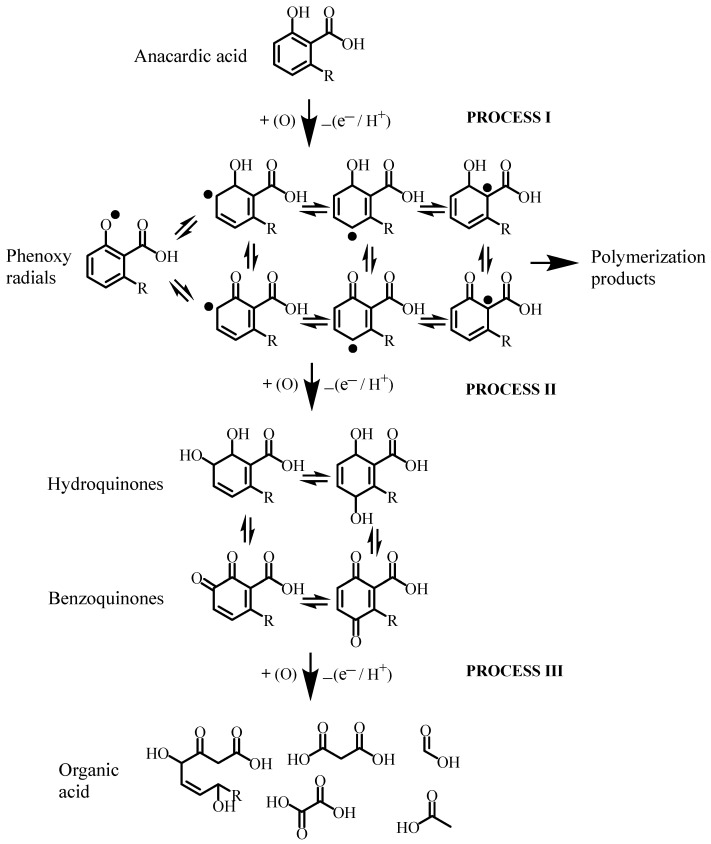
Schematic of the oxidation reactions of anacardic acid to low-molecular-weight organic acids and polymeric material.

Figure 5 shows a diagram of the reactions that take place during the electrochemical oxidation process of anacardic acid by applying the cyclic voltammetry potentials described above. The first electrochemical oxidation reaction (process I) occurs in the phenol group and is converted into phenoxyl radical, which is converted to hydroxyquinone or benzoquinone by a second electrochemical oxidation (process II). Phenolic radicals can also react by nucleation processes that lead to oligomerization or polymerization to become oligomers or polymers (process IV). Some works have proposed that the oligomers or polymers formed can undergo oxidations that disintegrate them to CO_2_ and H_2_O [23]. However, in the experiments carried out, no disintegration of the polymer was observed, and at the end of the electro-oxidation process, an ochre-colored material was observed at the bottom of the cell. Finally, hydroquinones and benzoquinones of anacardic acid undergo cleavage as a result of electro-oxidation processes with the consequent opening of the phenolic ring and molecular breakage and the generation of low-molecular-weight organic acids such as oxalic acid, citric acid, and acetic acid, among others.

**Figure 6:**
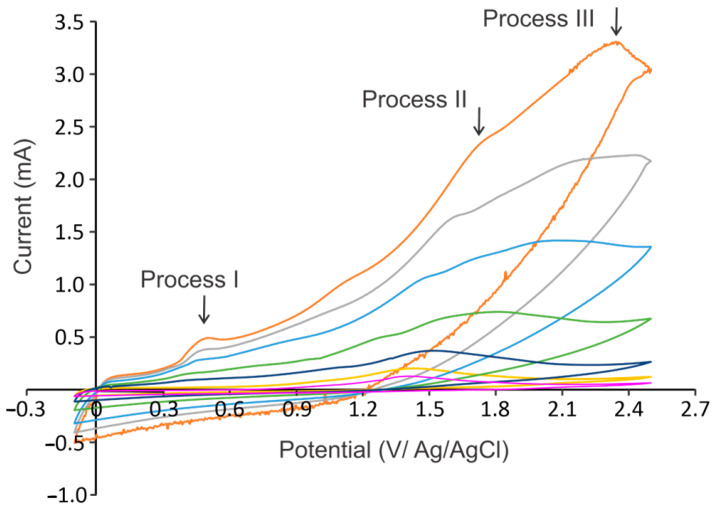
Cyclic voltamperometry of anacardic acid recorded with scan rates from 20 to 1000 mV/s.

On the other hand, it was observed that by increasing the voltametric scan rate, the oxidation processes shift to more positive potentials; so, the nucleation process is not evident when voltammograms with a scan rate higher than 20 mV/s are performed. However, precipitate is observed in small quantities; so, it can be inferred that polymerization takes place in very low quantities. Figure 6 shows the voltammograms of anacardic acid recorded at scan rate of 20 to 1000 mV/s. It can be observed that the electrochemical oxidation processes shift to more positive potentials as the scan rate increases. Additionally, it is observed that the currents recorded in the cycles rise as the scan rate increases; so, the current intensities of the oxidation peaks (*Ip*) increase. The study of the influence of the scan rate on the electrochemical behavior of the oxidation processes was determined by establishing the relationship of the peak potentials (*Ep*) and peak currents (*Ip*) versus the scan rates.

**Figure 7:**
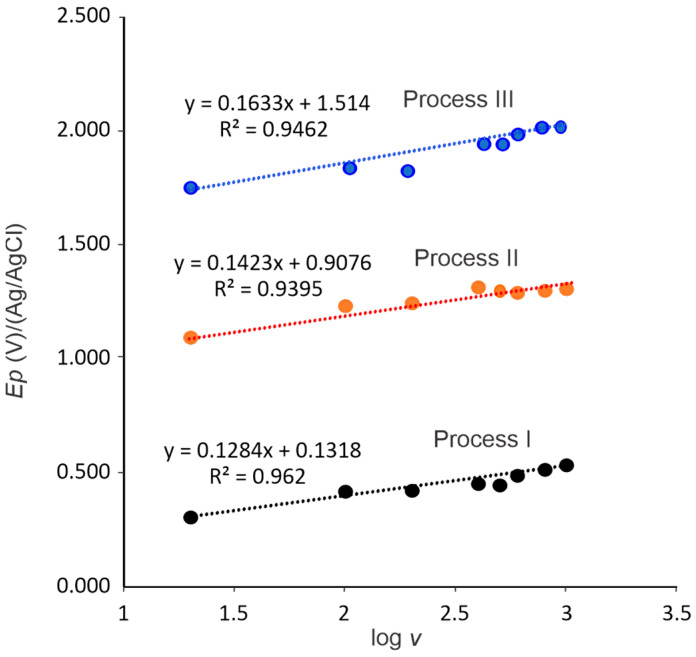
Correlation graphs of peak potentials (*Ep*) as a function of the logarithm of the scan rate (*log v*).

Figure 7 shows a graph of the oxidation peak potentials (*Ep*) versus the logarithm of the scan rates (*log v*). It can be observed that the peak potentials have a linear relationship with respect to the logarithm of the scan rates, establishing the equation $Ep\left(V\right)=b\left(intercet\right)+m\left(slope\right)log\;v$, with R^2^ of 0.962, 0.939 and 0.946 for processes I, II, and III, respectively. It has been established that in irreversible electrochemical reactions, the peak potential varies with the scan rate according to the Laviron equation [29]:

(1) $$Ep=E^{0}+\left(\frac{2.303RT}{\alpha nF}\right)\log\left(\frac{RTK^{0}}{\alpha nF}\right)+\left(\frac{2.303RT}{\alpha nF}\right)logv$$

where *E*^0^ is the standard formal potential, *R* is the gas constant (8.314 J mol^−1^ K^−1^), T is the temperature (K), *α* is the transfer coefficient, *n* is the number of transferred electrons, *F* is the Faraday constant (96.485 C mol^−1^), *K*^0^ is the standard heterogeneous rate constant of the reaction, and *v* is the scan rate.

The charge transfer coefficient (α) is a parameter that describes the ease of movement of electrons between the working electrode and the electro-active species and can be calculated using the Bard–Faulkner equation [30]:

(2) $$\propto =\frac{47.7}{E_{p}+E_{\frac{p}{2}}}$$

$E_{\frac{p}{2}}$ is the potential when the peak current is at half. The calculated electron transfer coefficient (α) values were 0.414, 0.397, and 0.407 for oxidation processes I, II, and III. From the α value, the *n* values were calculated. Thus, the values of the number of electrons transferred in each oxidation reaction (*n*) were 0.960 (~1.0) for process I, 0.976 (~1.0) for process II, and 0.941 (~1.0) for process III, indicating that one electron transfer is involved in all three oxidation processes. The *α* values suggest that electron transfers between anacardic acid and the electrode exhibit moderately fast kinetics. Furthermore, its slight asymmetry (~5.0) suggests the possible formation of intermediates and/or the influence of adsorption on the electrode surface. This fact is consistent with the mechanism presented in Figure 5, where the formation of intermediate states is presented.

Additionally, the intercept *m* allows the *K*^0^ value to be calculated once the *E*^0^ value has been determined by extrapolation of the curve to the vertical axis with *v* = 0. The *K*^0^ values calculated were 0.340 cm s^−1^, 0.104 cm s^−1^, and 0.241 cm s^−1^ for oxidation processes I, II, and III. This parameter is related to the rate of electron transfer and the calculated values indicate that electron transfer is faster in process I, being approximately three times greater than in process II and about 1.5 times greater than in process III. In addition, process III’s is greater than process II’s by a proportion close to 2.5 times. The values are in an intermediate range, which represents a moderate rate of electron transfer.

Taking into account that the values of *α* and *K*^0^ are related to the rate of electron transfer between the anacardic acid and the electrode, the values of these parameters suggest that electron transfer does not limit the electrochemical oxidation processes and that the processes can be controlled by diffusion or adsorption. This is corroborated by the study of the relationship between *Ip* and the scan rate.

**Figure 8:**
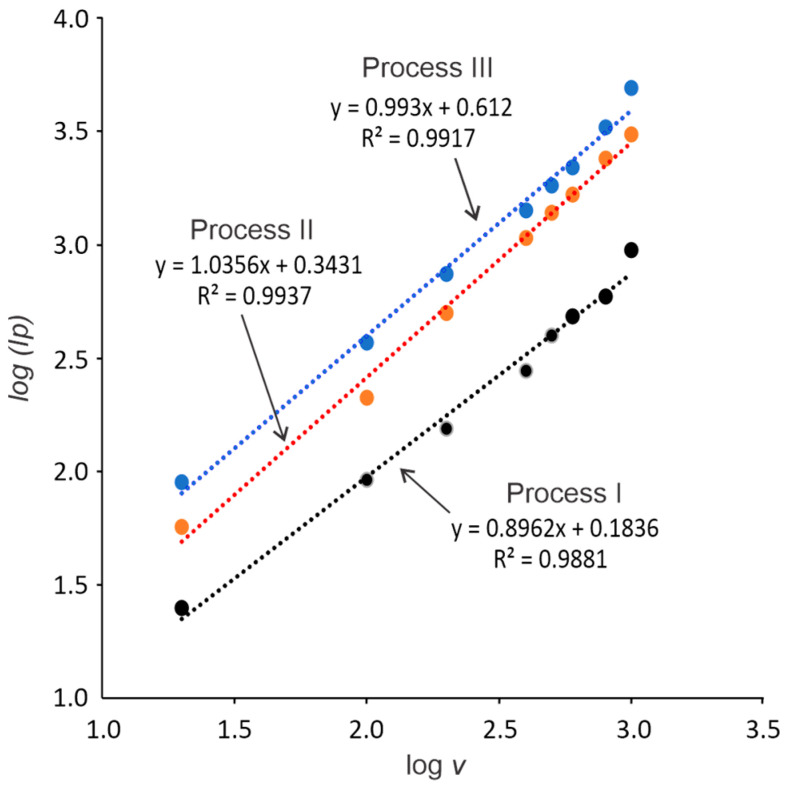
Correlation graphs of logarithm of peak currents (*log Ip*) as a function of the logarithm of the scan rate (*log v*).

Figure 8 presents the logarithm of the peak currents (*log Ip*) versus the logarithm of the scan rate (*log v*). It can be seen that there is a linear proportion relationship; *log Ip (mA) = b(intercet) + m(slope) (log v)*. The R2 values are 0.988 for process I, 0.993 for process II, and 0.991 for process III, indicating a high correlation between *log Ip* and *log v.* In addition, slope values (*m*) of 0.896, 1.035, and 0.993 are obtained for processes I, II, and III, respectively. The linear relationship with a slope close to 1.0 suggests that the processes are controlled by diffusion–adsorption [31]. This result is consistent with that suggested by the values of *α* and *K*^0^. With an increasing scan rate, electron transfer increases with a consequent increase in the recorded current, being limited by transport processes and the adsorption of anacardic acid.

**Figure 9:**
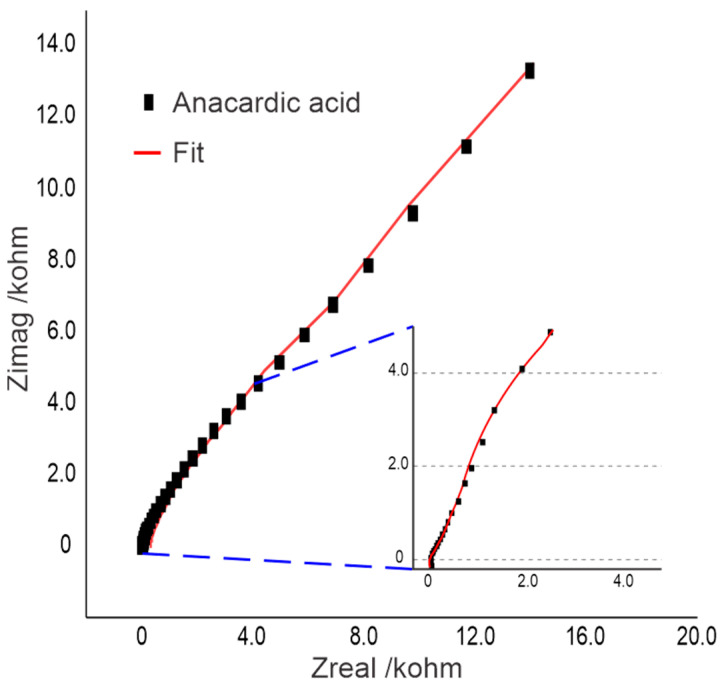
Nyquist plot of impedance spectroscopy of anacardic acid on a carbon electrode.

The analysis carried out by electrochemical spectroscopy is presented in Figure 9 by a Nyquist graph. It can be observed that when magnifying in the high-frequency zone, an inductive process is observed, which is suggested by values below zero. In addition, in the magnification, depressed semicircular curves are observed in the signals. The partial semicircles can be related to the Faradaic current of the oxidative processes of the anacardic acid molecules and superficial capacitive phenomena. In addition, impedance grows in an almost linear manner with the imaginary component, suggesting a diffusive response. The red fitting curve represents the trend of the experimental data well, which indicates that the equivalent model used fits well.

**Figure 10:**
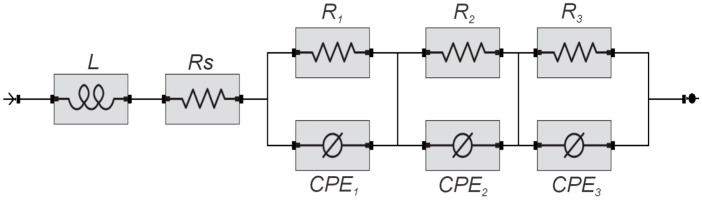
Equivalent circuit model of electrochemical oxidation of anacardic acid on a carbon electrode.

The equivalent circuit consists of an inductor element L connected in series to a resistor Rs followed in series by three systems composed of pairs of constant phase elements (*CPE 1*, *CPE 2*, and *CPE 3*) connected in parallel with their resistors (*R1, R2,* and *R3*). The equivalent circuit model corresponds to *L-Rs-[(R1/CPE1)-(R2/CPE2)-R1/CPE3)]*. The representation of the equivalent circuit is presented in Figure 10.

The equivalent circuit is formed by an inductor element (*L*) that is related to the porosity of the carbon electrode used in the electrochemical treatments (1.199 × 10^−4^ H). The resistance of the solution is represented as *Rs* and has a value of 17.189 ohms. The charge transfer resistances of the oxidation processes are represented in elements *R1*, *R2*, and *R3* with values of 2.118 × 10^−3^ ohms, 23.676 ohms and 3.765 ohms, respectively. These resistances accompany the constant phase elements that involve the oxidation reactions and capacitive phenomena in *CPE1* (0.612 S s^a^), *CPE2* (9.385 × 10^−5^ S s^a^) and *CPE3* (4.008 × 10^−5^ S s^a^). Additionally, *CPE* elements are commonly used in processes on non-homogeneous surfaces that present roughness and porosity defects [32,33].

## 3. Materials and Methods

### 3.1. Reagents and Solutions

The reagents were purchased from Merck and Aldrich. Deionized water with (18 MΩ cm^−1^) was used to prepare all solutions and synthesis reactions. The reagents used were sodium hydroxide (NaOH; Aldrich, 99.9%), methanol (CH_3_OH; Merck, 99.8%), calcium hydroxide (Ca(OH)_2_; Merck, 99.9%), ethyl acetate (C_4_H_8_O_2_; Merck, 99.8%), anhydrous sodium sulfate (Na_2_SO_4_; Merck, 99.0%), and chlorohydric acid (HCl; Aldrich, 37%).

Anacardic acid was extracted from cashew nut shell liquid (CNSL) obtained from the processing of cashew nuts (*Anacardium occidentale* Yucao Ao3 variety) and following the previously reported chemical method [34]. Extraction was performed using methanol as the solvent to ensure the purity and quality of the extracted compound. CNSL was obtained from fresh and dried shells through mechanical extraction at room temperature using an oil press machine (US Solid brand). Then, 100 g of CNSL was dissolved in 400 mL of a 5% methanol solution, and 50 g of calcium hydroxide was added. The resulting calcium anacardate precipitate was filtered and washed with water. This compound was then heated at 45 °C for 3 h and suspended in hydrochloric acid (37%), and the anacardic acid was extracted using ethyl acetate, followed by drying with anhydrous sodium sulfate. The extracted anacardic acid was purified by recrystallization in a cold methanol solution. Anacardic acid was identified using infrared spectroscopy, while its purity was determined through UV–vis spectroscopy and high-performance liquid chromatography (HPLC) [34]. For electrochemical oxidation treatment, extracted anacardic acid was used; the specific distribution of the unsaturation levels in the alkyl side chain (monoene, diene, triene) was not determined in this study.

### 3.2. Electrochemical Measurements and Spectroscopic Study

The electrolyte solution for the electrochemical experiments consisted of 0.1% NaOH in methanol and 0.1% anacardic acid. This solvent–electrolyte combination was selected for its ability to dissolve anacardic acid and facilitate redox reactions efficiently. Electrochemical experiments were conducted in a three-electrode cell configuration. A glassy carbon electrode (0.4 cm diameter) was used as the working electrode due to its wide potential window and chemical stability. A platinum plate served as the counter electrode, and a silver–silver chloride reference electrode (Ag/AgCl) was used as the reference electrode. Before each experiment, the glassy carbon electrode was polished using alumina slurry and rinsed thoroughly with deionized water to ensure reproducible results. The electrochemical oxidation of anacardic acid was carried out using cyclic voltammetry (CV) and electrochemistry impedance spectrometry (EIS). The potentiostat/galvanostat (Gamry ref. 1010E controlled with Gamry Framework software v.7.0) was operated under ambient conditions. Cyclic voltammetry experiments were carried out at scan rates ranging from 20 to 1000 mV/s, covering a potential window of −0.1 V to 2.5 V.

Electrochemical impedance spectroscopy was performed at a frequency range of 50 mHz to 20 KHz and an AC voltage of 10 mV rms with a cell equal to that used for cyclic voltammetry and using the same potentiostat. To perform the impedance spectroscopy analysis, the Echem Analyst 2.0 software was used. Characterization was carried out by FTIR-ATR spectroscopy performed with a Spectrum-two from a Perkin-Elmer spectrometer, with an ATR (Attenuated Total Reflectance) accessory. To record the spectra, a resolution of 4 cm^−1^, a wavenumber range of 400 cm^−1^ to 4000 cm^−1^, and 100 scans were used.

## 4. Conclusions

The feasibility of electrochemical oxidation of anacardic acid, derived from cashew shell liquid (CNSL), for the generation of organic acids and polymeric materials was demonstrated. Using cyclic voltammetry (CV), electrochemical impedance spectroscopy (EIS) and FTIR spectroscopy, multiple oxidation processes involving the formation of phenoxide, hydroxyquinone and benzoquinone radicals were identified, leading to ring opening and the production of low-molecular-weight organic acids (propionic, formic, oxalic, lactic and acetic acids) confirmed by HPLC. The experiments showed that the oxidation of anacardic acid is irreversible, with charge transfer coefficients between 0.397 and 0.414, suggesting a diffusive–adsorptive control at the electrode–solution interface. Furthermore, a polymerization process was observed, evidenced by the formation of a solid material after electrochemical treatment. FTIR analysis confirmed modifications in the functional groups of anacardic acid, validating its conversion into oxidized products and polymers. The results highlight the potential of electrochemistry as a sustainable route for the transformation of biomass into high-value-added compounds, minimizing the dependence on petrochemical sources and promoting the efficient use of agro-industrial waste.

## Data Availability

Data is contained within the article.
